# National soil hydrologic groups map for environmental applications using data-driven and expert-based methods

**DOI:** 10.1038/s41597-025-05853-5

**Published:** 2025-09-29

**Authors:** Brigitta Szabó, Ronald András Kolcsár, János Mészáros, Annamária Laborczi, Katalin Takács, Gábor Szatmári, András Makó, Kálmán Rajkai, Balázs Benyhe, Károly Barta, László Pásztor, Zsófia Bakacsi

**Affiliations:** 1 Institute for Soil Sciences, HUN-REN Centre for Agricultural Research, Budapest, Hungary; 2 National Laboratory for Water Science and Water Security, Budapest, Hungary; 3 Lower Tisza District Water Directorate, Szeged, Hungary; 4 Department of Physical and Environmental Geography, University of Szeged, Szeged, Hungary

**Keywords:** Environmental impact, Hydrology

## Abstract

Regional and national 3D soil hydraulic maps enhance understanding of soil hydraulic properties, essential for environmental assessments. However, data aggregation is often necessary in large-scale models to facilitate the modelling of complex soil characteristics. This study presents a soil hydrologic groups map for Hungary, derived through k-means clustering and expert-based rules. Clustering was applied to the 100 m resolution 3D HU-SoilHydroGrids database, considering eight hydraulic parameters across six depths. The accuracy of these maps is limited for rare soil types with extreme characteristics due to their small spatial extent and sparse representation in national datasets. To account for these underrepresented soil types, we refined each statistics-based cluster using expert-based rules incorporating soil profile depth, genetic type, electrical conductivity, and exchangeable sodium content. The final classification includes 68 soil hydrologic groups, defined by distinct hydraulic properties, such as van Genuchten parameters to describe water retention, and saturated hydraulic conductivity. This national map supports country-wide hydrological modelling, environmental management, and agricultural planning in Hungary by enabling consistent treatment of similar soils.

## Background & Summary

Soil hydraulic groups – classifications of soils by their water transmission and retention characteristics – are foundational in water-related disciplines. Grouping soils by their hydraulic behaviour simplifies the representation of complex soil properties in models and management plans. Soil hydraulic groups enhance runoff and flood predictions by classifying soils based on their hydraulic behaviour. Models like the United States Department of Agriculture (USDA) Curve Number method^1^ assign different runoff potentials to soil groups, helping estimate infiltration versus runoff. Accurate classification improves flood forecasting, soil erosion modelling, and watershed management. It also supports environmental conservation by identifying areas prone to erosion, flooding, or leaching risks^2^.

Many widely used hydraulic group systems were originally derived from expert knowledge and field observations. For example, the USDA’s Hydrologic Soil Groups were first assigned based on measured rainfall-runoff and infiltration data, and later by soil scientists’ judgment following published guidelines^3^. In practice, soil survey experts consider properties like texture, depth, drainage, and permeability to place a soil in a group. Expert classification ensures that qualitative factors (like presence of a hardpan or a high water table) are accounted for. This system uses four base groups (A-D) for categorisation, as well as additional three dual groups in case of soils where drainage is feasible (A/D, B/B, C/D)^1^. This rule-based method relies on threshold criteria (e.g. sandier soils with high permeability are assigned to Group A; clayey or shallow soils go into Group D).

Some frameworks use decision trees built by experts – for instance, the UK’s Hydrology of Soil Types (HOST) system classifies soils based on their hydrological response and dominant water pathways (vertical infiltration, lateral flow, surface runoff). The soils are grouped with expert-based decision rules considering soil texture, soil structure, depth, presence of impermeable layers, groundwater levels, and geology^4–6^. This way HOST combines soil surveys and geological maps, using expert rules and hydrological interpretations. These expert-based methods are transparent and grounded in soil science theory, though they can be limited by subjectivity and coarse groupings.

To improve objectivity, researchers increasingly apply statistical clustering to derive soil hydraulic groups from measured or simulated data. Unsupervised learning methods, most often k-means clustering, have been used to partition soils into homogeneous hydraulic clusters^7–9^. For example, Wösten *et al*.^10^ computed key functional soil characteristics (e.g. water storage capacities, hydraulic conductivity) for hundreds of soil mapping units, then used k-means clustering to group them into an optimal six “soil hydraulic units” with similar hydrologic behaviour. Such data-driven grouping can reveal natural clusters in continuous properties like water retention curve parameters or saturated hydraulic conductivity. Similarly, Groenendyk *et al*.^7^ performed numerical hydrologic simulations across the USDA texture spectrum and applied k-means clustering to the model outcomes, deriving new soil classes based on actual infiltration and drainage response rather than just texture labels. This approach showed that traditional texture classes can be poor predictors of hydrologic response, and clustering by modelled behaviour yields more accurate groupings. The advantage of clustering methods is consistency and adaptability, they can incorporate large soil databases and objectively identify group boundaries based on data patterns, ensuring that soils within a cluster have statistically similar hydraulic profiles.

Environmental models often require aggregated input data for specific modelling units. These units are typically defined using land use, slope, and soil classifications based on soil type or texture, as seen in applications like the Soil and Water Assessment Tool^11^. However, when the goal is to distinguish soils based on their hydrological properties, soil types or soil texture alone might not be sufficient for optimally defining soil hydrologic groups. Moreover, maps of soil hydrologic groups are not frequently available. For global applications, the HYSOGs250m map exists^12^. In Hungary, a qualitative soil water management category map has been available at a 1:500000 scale, featuring nine categories and seventeen variants. This classification was established through expert rules based on factors such as field capacity, wilting point, available water content, infiltration rate, saturated hydraulic conductivity – these were delineated based on basic soil properties –, and soil texture variations^13^.

In Hungary, national 3D soil hydraulic maps are available at a 100 m resolution. However, the use of these detailed maps is not always feasible for national, daily-based environmental simulations, such as those performed by the Operational Drought and Water Scarcity Monitoring System (DWMS, https://vizhiany.vizugy.hu/). Therefore, the aim of our work was to aggregate the information available from the 100 m resolution maps by developing a quantitative soil hydrologic groups map for Hungary, rather than simply reducing the map’s resolution. This was achieved by i) using the newly available national 3D soil hydraulic maps (HU-SoilHydroGrids), the basic soil maps (DOSoReMI.hu) and information on salt-affectedness at 100 m resolution; and ii) by applying both statistical clustering and expert-based rules.

## Methods

### Input data

For the preparation of the soil hydrologic groups map, we used the national, 3D soil hydraulic maps (HU-SoilHydroGrids)^14^, maps of basic soil properties (DOSoReMI.hu, https://dosoremi.hu/)^15^, maps of salt affected soils^16^, as well as the Hungarian Detailed Soil Hydrophysical Database, called MARTHA (Hungarian acronym of the dataset’s name)^17^. All the input soil maps have 100 m spatial resolution and cover the territory of Hungary.

The HU-SoilHydroGrids dataset holds information about water content at saturation, field capacity and wilting point, saturated hydraulic conductivity, and van Genuchten parameters for the description of the moisture retention curve down to 2 m depth for six standard depth layers – defined by the Global Soil Map (GSM).

The DOSoReMI.hu maps include soil chemical, physical and taxonomical information with national coverage at 100 m resolution for the above-mentioned GSM standard depth layers. From this dataset we included the soil type and rooting depth in the analysis.

The map of the salt-affected soils includes information on the electrical conductivity (EC), exchangeable sodium percentage (ESP) and pH for the 0–30 and 30–100 cm soil depth, at a 100 m resolution.

MARTHA is a point soil profile dataset with measured soil chemical, physical, hydrological, and taxonomical information. It holds measured data of 15142 soil horizons belonging to 3970 soil profiles.

### Deriving soil hydrologic groups

**Fig. 1:**
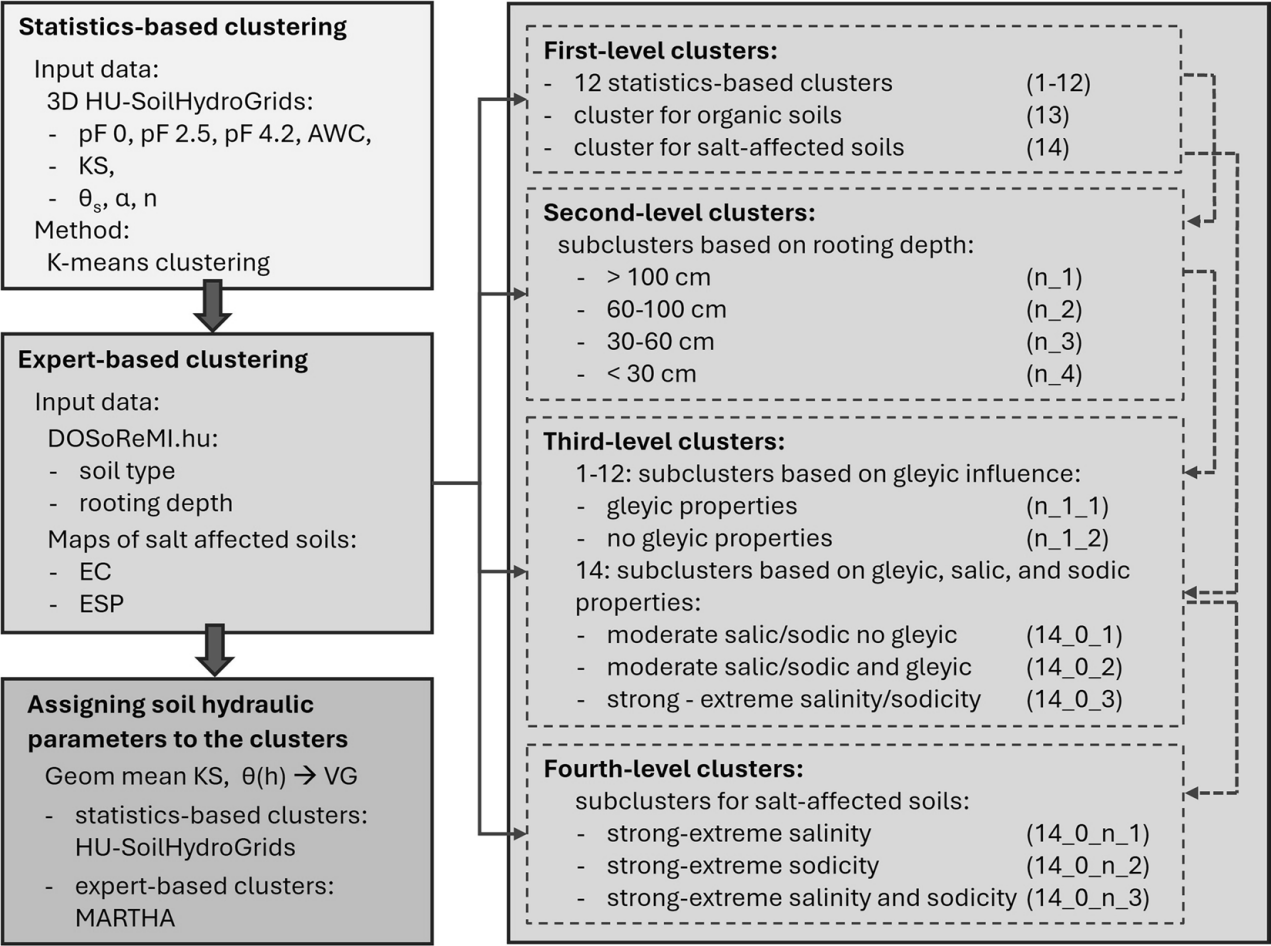
Workflow for deriving soil hydrologic groups and their hydraulic parameterization.

The soil hydrologic groups were derived in two steps. First statistics-based clustering was applied on the soil hydraulic maps. Then, these clusters were further refined using expert-based rules to differentiate soils that are underrepresented in the soil hydraulic maps but need to be distinguished due to their specific physico-chemical behaviour. Figure 1 shows the main steps of the clustering process.

#### Statistics-based clustering

A k-means clustering algorithm was used to delineate soil hydraulic groups. The clustering was based on eight soil hydraulic parameters of six GSM soil depths (0–5, 5–15, 15–30, 30–60, 60–100 and 100–200 cm). The eight parameters were water retention at saturation (pF 0), field capacity (pF 2.5), wilting point (pF 4.2), available water capacity (AWC) – derived from field capacity and wilting point –, saturated hydraulic conductivity (KS) and parameters of the van Genuchten model^18^ (θ_s_, α and n) (Eq. 1).

1 $$\theta \left(h\right)={\theta }_{r}+\frac{{\theta }_{s}-{\theta }_{r}}{{\left[{1+\left(\alpha \left|h\right|\right)}^{n}\right]}^{m}}$$

where *θ*(*h*) is the water content of the soil (cm³ cm^−3^) at a given matric potential value (cm of water column); *θ*_*r*_ is the residual water content (cm³ cm^−3^); *θ*_*s*_ is the saturated water content (cm³ cm^−3^); and *α* (cm^−1^), *n* (−), and *m* (−) are fitting parameters. Parameter *m* was set equal to 1-1/n, *θ*_*r*_ was set to 0^19^.

The water retention curve could be more accurately described using bimodal models^19,20^, such as those by Ross and Smettem^21^, or Durner^22^ or Romano *et al*.^23^, among others. However, these models require fitting more parameters than unimodal models, which in turn demands more than five measured water retention-pressure head (θ-h) pairs^19^. This data requirement reduces the number of samples available for predicting and mapping parameters of soil water retention function, thereby limiting their broader applicability. For this reason, the van Genuchten model was chosen to describe soil water retention in the development of the HU-SoilHydroGrids dataset, as it is a widely applicable unimodal model.

The distribution of the variables was evaluated using skewness-kurtosis test. Variables with non-normal (or close to normal) distribution were transformed^8^. For KS and parameter α, a log_10_ transformation was applied. For the values of n, log_10_ (n-1) transformation was applied.

The k-means clustering was performed on the pixel values of the map, using the eight hydraulic properties – mentioned above – of the six soil depths, resulting in 48 input variables. This input data were standardized, and the optimal number of clusters was determined in the clustering. The analysis identified twelve distinct clusters.

#### Expert-based clustering

The statistics-based twelve clusters had three important shortcomings, (i) within the clusters, there was some heterogeneity in terms of soil organic carbon content, EC, ESP, and soil types – all of which indicate specific physico-chemical properties that are important to distinguish during environmental analysis and assessments; (ii) the analysis considered mapped soil hydraulic data derived by an ensemble method, thus the input data was not representing soil profiles with extreme soil chemical or physical properties; and (iii) lacking information on whether soil depth is restricted by bedrock. These shortcomings necessitated further refinement of the clusters through expert-based grouping. The Hungarian genetic soil types include information on several physical and chemical soil properties^24–26^, therefore, the soil type map was used to define expert-based groups with specific hydrological properties. Additionally, rooting depth, EC, and ESP values were considered in this step of the clustering.

##### Expansion of first level clusters with organic soils and salt-affected soils

The statistics-based primary clusters were expanded by two expert-created categories: organic soils and salt-affected soils, due to their specific soil hydrological response.

Organic soils were extracted from the 12 statistics based clusters, based on their soil type, i.e., soils with an organic horizon^27^, which are soil types 320, 340 and 350 according to the Hungarian classification system^25,26^. These soils were moved to a separate cluster 13, but only if by their EC and ESP values they did not meet the criteria of salt-affected soils.

Salt-affected soils were also manually moved to a separate cluster – cluster 14 – selected from the twelve statistics-based clusters. These include any soils that have:

- salt-affected soil types, i.e. have natric or salic horizon^27^, which correspond to soil types with codes between 220–290 and 303 according to the Hungarian classification system, or
- an EC value equal to or higher than 2 dS·m^−1^, or
- an ESP value in the upper 100 cm equal to or higher than 30%.

The above-mentioned thresholds were identified based on FAO classification to identify intensity of salt problems in soil, applying FAO threshold for salinity (EC) and the definition by Abrol *et al*. for sodicity (ESP)^28^. In summary, soils with moderate or stronger salinity and sodicity were classified under cluster 14.

##### Second-level clusters based on rooting depth

With the exception of cluster 13 – which includes the organic soils – and 14 – with salt-affected soils – each primary class was further divided based on rooting depth.

Soils with a rooting depth deeper than 100 cm, or the ones whose genetic soil type does not indicate shallow depth (i.e. soil types other than 10, 70, or 91 in the Hungarian soil classification system^25,26^), were placed in the first secondary cluster and labelled as “n_1.”

Shallow soils (soil types 10, 70, and 91) were classified as follows: those with a rooting depth between 60 and 100 cm as “n_2,” those between 30 and 60 cm as “n_3,” and those with a rooting depth of less than 30 cm as “n_4.”

For organic soils (cluster 13) and salt-affected soils (cluster 14), no further differentiation was applied; therefore, their second-level clusters were designated as 13_0 and 14_0, respectively.

##### Third-level clusters based on gleyic influence

Secondary clusters belonging to clusters 1–12 that were not classified as shallow (i.e., clusters 1_1, 2_1, …, 12_1) were further divided into third-level clusters based on the presence of a gleyic horizon. Soils with gleyic properties, as identified by the Hungarian soil classification system (soil types coded 280–330^25,26^), were categorized as “n_1_2”, while those without gleyic properties were labelled as “n_1_1”.

Within cluster 14, soils with moderate salinity and/or sodicity – according to the FAO classification – but no gleyic properties were classified as 14_0_1. Soils with moderate salinity and/or sodicity, that also exhibited gleyic properties were assigned to 14_0_2. Additionally, an extra cluster, 14_0_3, was defined for soils with strong, very strong, or extreme salinity and/or sodicity.

##### Fourth-level clusters for salt-affected soils

The third-level clusters of salt-affected soils were further divided into fourth-level clusters based on their EC and ESP values to determine whether a soil has moderate or stronger salinity, sodicity, or both. The following rules were applied to define the fourth level:

- if EC was equal to or higher than 2 dS·m^−1^ and ESP was lower than 30% (strong or very strong or extreme salinity), it was assigned to cluster 14_0_n_1,
- if EC was lower than 2 dS·m^−1^ and ESP equal to or higher than 30% (strong or extreme sodicity), it was assigned to cluster 14_0_n_2,
- if EC was equal to or higher than 2 dS·m^−1^ and ESP equal or higher than 30% (strong or very strong or extreme salinity and strong or extreme sodicity), it was assigned to cluster 14_0_n_3.

If EC, ESP, or both were unavailable, the information provided by the soil type was used to differentiate soils with strong salinity, sodicity, or both.

For the third-level clusters of non-salt-affected soils (subclusters of clusters 1–13), the fourth-level clusters were labelled as 1_1_1_0, 1_1_2_0, …, and 13_0_0_0, since these soils are neither salic nor sodic and therefore do not need to be distinguished based on EC and ESP.

After incorporating the expert-based rules, we finalized 68 clusters.

#### Assigning soil hydraulic parameters to the clusters

For the fourth-level clusters within clusters 1–12, we computed eleven water retention-pressure head pairs – at −0.001, −2.5, −10, −25, −100, −330, −500, −1000, −1500, −15000, −1500000 cm – for all pixels and each depth layer of the HU-SoilHydroGrids map, based on the mapped van Genuchten parameters. Then, for each fourth-level cluster and depth layer, we calculated the geometric mean of the water retention values at each pressure head values, as well as the saturated hydraulic conductivity. Finally, the van Genuchten model was fitted – i.e., parameters θ_s_, α and n (with θ_r_ fixed at 0) – to the geometric mean water retention-pressure head pairs for each cluster and depth layer. The resulting van Genuchten parameters, along with the geometric mean saturated hydraulic conductivity, describe the soil hydrological properties of the clusters.

The characteristic van Genuchten parameters and saturated hydraulic conductivity values for the subclusters of clusters 13 and 14 were computed using the MARTHA point dataset. First, we applied the expert-based rules to the dataset to differentiate samples belonging to subclusters of cluster 13 and 14. For this, we computed EC and ESP values for the MARTHA dataset using Eqs. [2, 3]^16,24,29^, based on the relevant, available soil properties.

2 $${EC}=1254.7\cdot \frac{{salt}}{1.2\cdot {K}_{A}}$$

3 $$\begin{array}{ccc}{ESP} & = & 4.3461\cdot {{pH}}^{2}-52.195\cdot {pH}+139.61\,{if\; pH}\ge 8\\ {ESP} & = & 0.001\,{if\; pH} < 8\end{array}$$

Then, we derived the characteristic van Genuchten parameters and soil hydraulic conductivity using the method described in the previous section. The key difference is that the characteristic van Genuchten parameters of the clusters were computed based on measured water retention–pressure head pairs, rather than the mapped values from HU-SoilHydroGrids, where these soils are underrepresented. This approach ensures that soil-type-specific hydraulic parameters are assigned to organic and salt-affected soils. While the unique physico-chemical properties of these soils may not be directly reflected in their hydraulic properties, incorporating information about their chemical characteristics allows for a more accurate definition during input data processing. This, in turn, enables better adjustments in modelling applications.

## Data Records

**Fig. 2:**
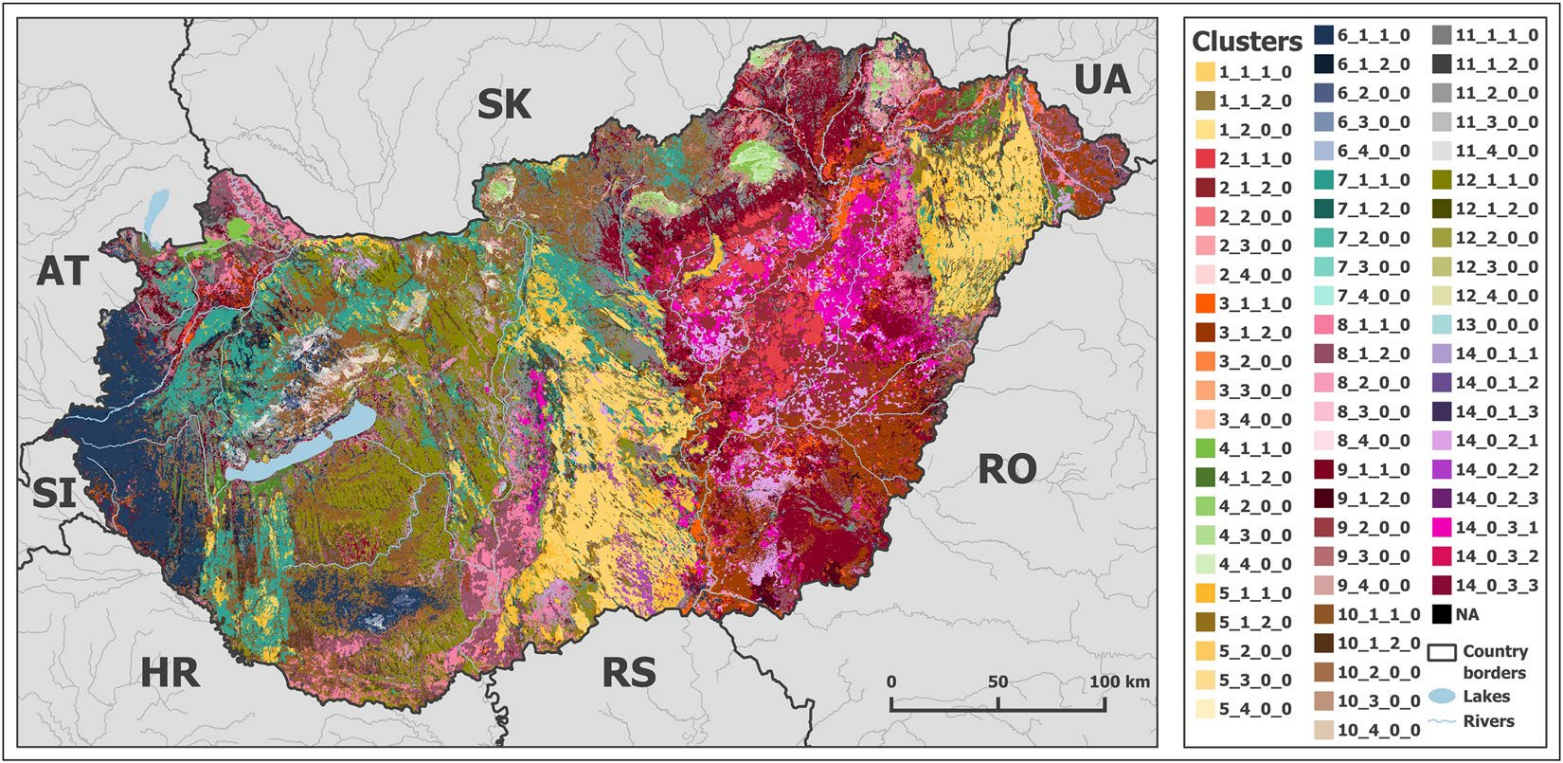
The newly developed national soil hydrologic groups map of Hungary.

The dataset is available on the Zenodo online repository^30^. The map is in GeoTIFF format with a coordinate system of the Hungarian Unified National Projection System (EOV/HD72 – ID: 23700 in the EPSG Geodetic Parameter Data set^31^, see https://epsg.io/23700). It has a resolution of 100 m and covers the entire area of Hungary. The meanings of the map codes in terms of cluster number and soil hydraulic properties are provided in a single CSV file. The units of the soil hydraulic properties are specified in the metadata XML file of the map. Figure 2 shows the soil hydrologic groups map.

## Technical Validation

**Fig. 3:**
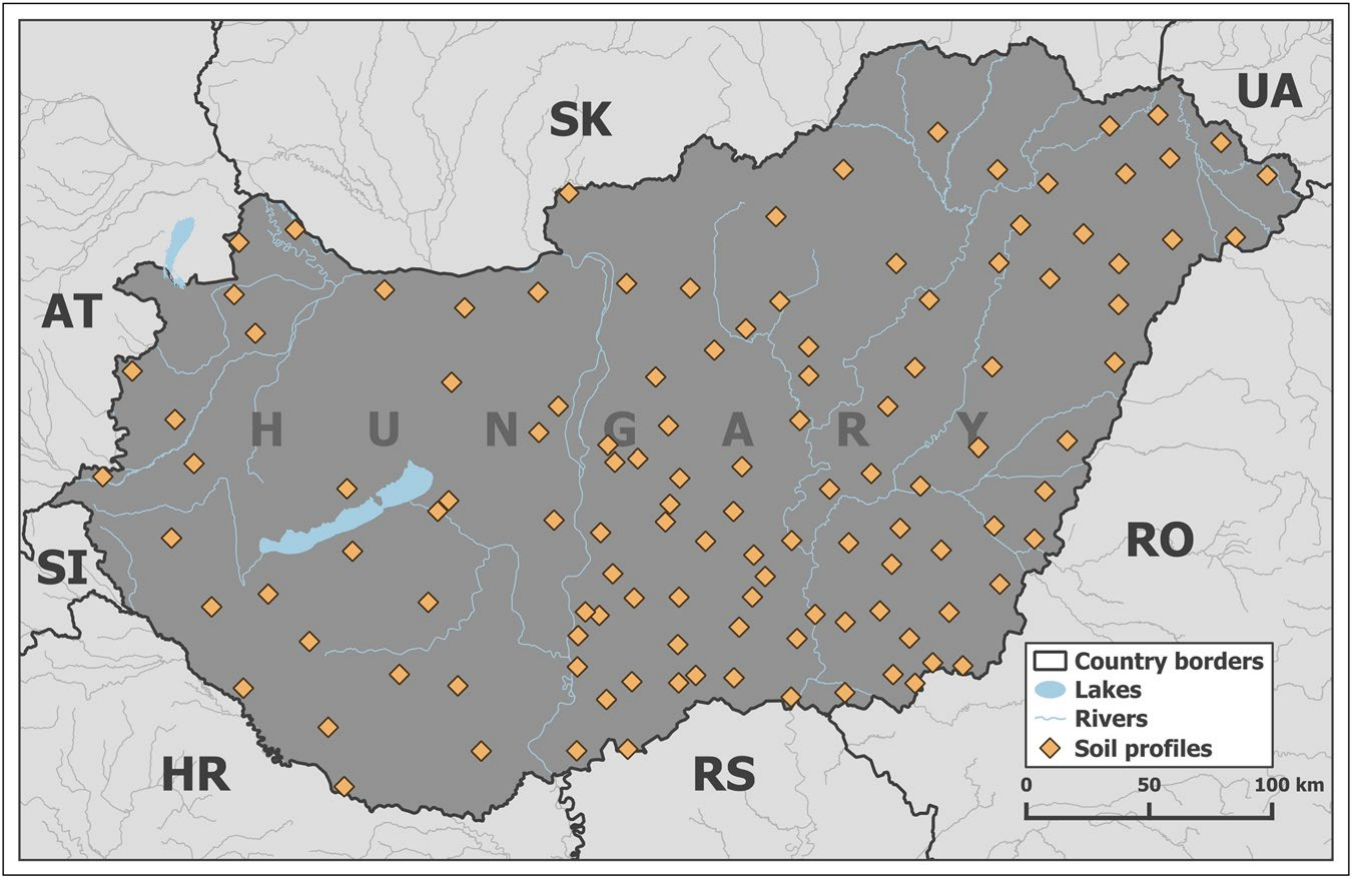
Location of the soil profiles of the Operational Drought and Water Scarcity Monitoring System (https://vizhiany.vizugy.hu/).

The validation of the soil hydrologic groups map was performed based on the measured point data of the Operational Drought and Water Scarcity Monitoring System. This dataset is independent of all input datasets that were used to prepare the soil hydrologic groups map. The monitoring system includes measured data on water retention and specific pressure head values, such as 0, −31.6, −100, −316, −15849, and −1584893 cm – i.e., pF 0, 1.5, 2, 2.5, 4.2, and 6.2 – of 726 soil layers belonging to 121 soil profiles (Fig. 3). Table 1 shows the descriptive statistics of the measured soil water retention data from the monitoring system.

**Table 1:** Descriptive statistics of the measured water retention data (cm³ cm^−3^) at different pressure head values – from pF 0 to pF 6.2 – of the Operational Drought and Water Scarcity Monitoring System.

Soil water retention* (cm³ cm^−3^)	N	Mean	Min	Max	Range	SD	SE	CV
pF 0	726	0.4597	0.3230	0.6290	0.3060	0.0575	0.0021	0.1251
pF 1.5	726	0.3942	0.2400	0.5900	0.3500	0.0451	0.0017	0.1144
pF 2	726	0.3447	0.0940	0.5790	0.4850	0.0842	0.0031	0.2442
pF 2.5	726	0.3188	0.0800	0.5490	0.4690	0.0903	0.0034	0.2832
pF 4.2	726	0.1240	0.0081	0.4640	0.4559	0.0664	0.0025	0.5361
pF 6.2	726	0.0203	0.0009	0.1100	0.1091	0.0142	0.0005	0.7008

*Soil water retention at 0, −31.6, −100, −316, −15849, and −1584893 cm pressure head values, respectively.

For the validation of the map, we computed the coefficient of determination (R^2^) (Eq. 4), mean error (ME) (Eq. 5), root mean square error (RMSE) (Eq. 6), and Lin’s concordance correlation coefficient^32^ (CCC) (Eq. 7).

4 $${R}^{2}=1-\frac{\mathop{\sum }\limits_{i=1}^{N}{({y}_{i}-{\hat{y}}_{i})}^{2}}{\mathop{\sum }\limits_{i=1}^{N}{({y}_{i}-\bar{y})}^{2}}$$

where *y*_*i*_ is the measured soil water content, *ŷ*_*i*_ is the predicted soil water content, $\bar{y}$ is the mean of the $y$ value, and *N* is the number of *y*_*i*_ and *ŷ*_*i*_ data pairs.

5 $${ME}=\frac{1}{N}\mathop{\sum }\limits_{i=1}^{N}\left({y}_{i}-{\hat{y}}_{i}\right)$$

6 $${RMSE}=\sqrt{\frac{1}{N}\mathop{\sum }\limits_{i=1}^{N}{\left({y}_{i}-{\hat{y}}_{i}\right)}^{2}}$$

7 $${CCC}=\frac{2\rho {\sigma }_{y}{\sigma }_{\hat{y}}}{{(\bar{y}-\bar{\hat{y}})}^{2}+{{\sigma }_{y}}^{2}+{{\sigma }_{\hat{y}}}^{2}}$$

where ρ is the correlation coefficient between the *y* measured and *ŷ* predicted soil water content, and σ_y_^2^ and σ_ŷ_^2^ are the corresponding variances.

We compared the performance of the soil hydrologic groups map with the HU-SoilHydroGrids maps to assess the uncertainty introduced by the aggregation of the soil hydrological data.

For validation, it was necessary to delineate the different soil depths of the map and the Operational Drought and Water Scarcity Monitoring System. To secure physical consistency between the soil hydraulic properties at different soil depths, interpolation was not applied. Instead, the mapped values were assigned to the monitoring system data based on the closest matching depth. (Table 2).

**Table 2:** Delineation of soil depth intervals between the sampling depth of the Operational Drought and Water Scarcity Monitoring System and the six Global Soil Map (GSM) standard depth layers of the soil hydrologic groups map.

Sampling depth of the Operational Drought and Water Scarcity Monitoring System (cm)	GSM standard depth layers of the soil hydrologic groups map (cm)
—	0–5
10	5–15
20	15–30
30, 45	30–60
60, 75	60–100
—	100–200

**Fig. 4:**
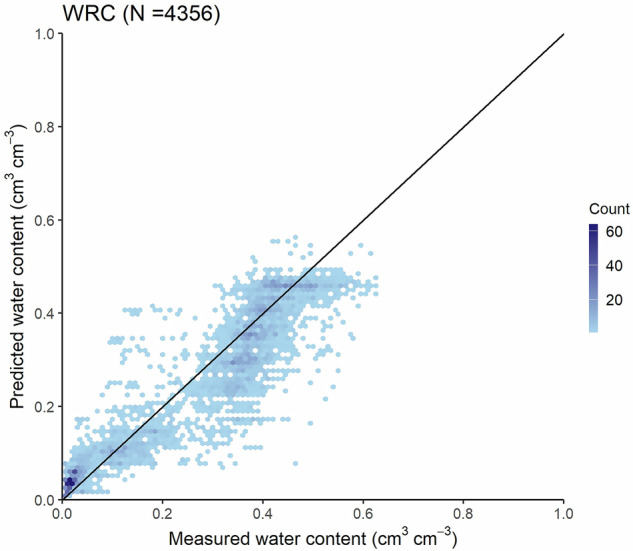
Accuracy of the soil hydrologic groups map based on the Operational Drought and Water Scarcity Monitoring System point dataset (number of water retention-pressure head pairs: 4356) for the entire water retention curve.

**Fig. 5:**
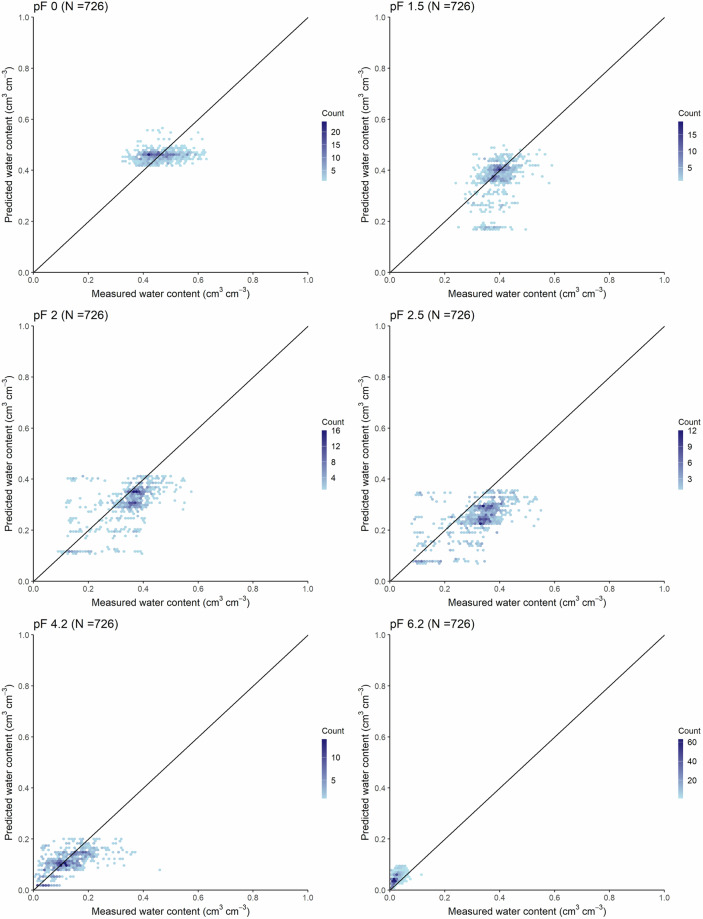
Accuracy of the soil hydrologic groups map by measured pressure head values – from pF 0 to pF 6.2 – based on the Operational Drought and Water Scarcity Monitoring System point dataset. pF 0 – pF 6.2: soil water retention at 0, −31.6, −100, −316, −15849, and −1584893 cm pressure head values, respectively.

Accuracy of the soil hydrologic groups map – along with the HU-SoilHydroGrids dataset – based on the Operational Drought and Water Scarcity Monitoring System point dataset is shown in Table 3 and Figs. 4, 5.

**Table 3:** Accuracy of the soil hydrologic groups map and HU-SoilHydroGrids maps in terms of water retention (cm^3^ cm^−3^) by pressure head values (from pF 0 to pF 6.2) and for the entire soil water retention curve (WRC) based on the Operational Drought and Water Scarcity Monitoring System point dataset.

Map	Soil hydraulic property*	R^2^	ME	RMSE	CCC	N	θ-h pairs
Soil hydrologic groups map	pF 0	0.036	0.002	0.057	0.117	726	—
	pF 1.5	0.137	0.025	0.074	0.307	726	
	pF 2	0.362	0.037	0.080	0.537	726	
	pF 2.5	0.377	0.073	0.102	0.419	726	—
	pF 4.2	0.352	0.014	0.055	0.520	726	—
	pF 6.2	0.338	−0.023	0.029	0.300	726	
	WRC	0.840	0.021	0.070	0.905	726	4356
HU-SoilHydroGrids	pF 0	0.090	0.002	0.055	0.218	726	—
	pF 1.5	0.226	0.009	0.069	0.410	726	—
	pF 2	0.584	0.015	0.061	0.752	726	—
	pF 2.5	0.607	0.050	0.077	0.665	726	
	pF 4.2	0.484	0.004	0.048	0.679	726	
	pF 6.2	0.451	−0.027	0.035	0.315	726	
	WRC	0.889	−0.013	0.057	0.939	726	4356

R^2^: coefficient of determination, ME: mean error, RMSE: root mean square error, CCC: Lin’s concordance correlation coefficient, N: number of samples, θ-h pairs: number of water retention-pressure head value pairs.

*pF 0 – pF 6.2: soil water retention at 0, −31.6, −100, −316, −15849, and −1584893 cm pressure head values, respectively.

The prediction of pF 0 has the highest uncertainty, as indicated by the R^2^ and CCC values. This is due to the fact that bulk density could not be included as a predictor in the computation of the HU-SoilHydroGrids maps. It is well-documented in the literature that bulk density is the most important predictor among basic soil properties for estimating soil hydraulic properties near saturation^33^, but it is often unavailable at national scale, as is the case for Hungary. The same reasoning applies to pF 1.5.

For pF 6.2, the uncertainty may stem from the type of model used to describe the soil water retention curve – namely, the van Genuchten model – which accurately describes water retention between pF 0 and 4.2 for unimodal soils but has lower accuracy in the dry range^34^, particularly pF 6.2, where overprediction is observed.

Water retention at pF 2, 2.5 and 4.2 had the highest accuracy in terms of R^2^ and CCC value. However, a systematic underprediction is evident, especially for pF 2.5. When assessing prediction performance across the entire matric potential range, the overall performance improves, having an RMSE of 0.070 cm^3^ cm^−3^. Nevertheless, the map’s limitations can be observed in Figs. 4, 5, where the aforementioned underprediction and overprediction are detected.

**Fig. 6:**
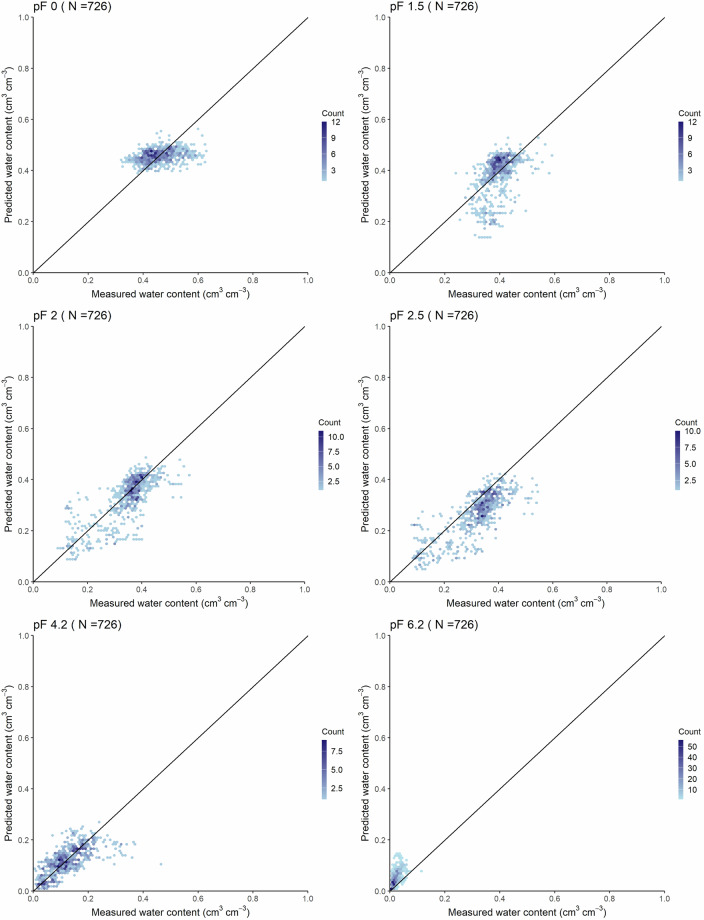
Accuracy of the HU-SoilHydroGrids maps based on the Operational Drought and Water Scarcity Monitoring System point dataset. pF 0 – pF 6.2: soil water retention at 0, −31.6, −100, −316, −15849, and −1584893 cm pressure head values, respectively.

Compared to the performance of HU-SoilHydroGrids (Table 3 and Fig. 6), all pF values show lower accuracy in the soil hydrologic groups map, which was expected due to the aggregation. Therefore, users must consider the impact of aggregation on the uncertainty of the mapped values.

Despite the reduction in accuracy due to aggregation, the soil hydrologic groups map remains highly valuable for many applications. Aggregation simplifies complex soil property datasets, making them more accessible for large-scale modelling efforts where computational efficiency is a priority. While high-resolution datasets provide greater precision, they are often impractical for regional or national-scale simulations due to their complexity and data processing requirements. The grouped approach allows for consistent treatment of soils with similar hydrological behaviour, ensuring reliable input data for hydrological and environmental models. This makes the map a crucial tool for decision-making in water resource management, land use planning, and climate impact assessments, where broad-scale patterns are more important than fine-scale variations.

In the future, it will be worthwhile to analyse if a computed bulk density could reduce the uncertainty in predicting soil hydraulic properties close to saturation. Another important aspect to consider for future aggregation methods is the selection of soil hydraulic properties used in the aggregation process. If only a few specific soil hydraulic properties are required, more specific and accurate groups could be derived.

## Usage Notes

The soil hydrologic groups map includes cluster codes, while the accompanying CSV file provides the soil hydraulic parameters – including the van Genuchten model parameters and saturated hydraulic conductivity – for all clusters at soil depth of 0–5, 5–15, 15–30, 30–60, 60–100, and 100–200 cm. Specific water retention values at a desired matric potential value – e.g. such as the wilting point – can be computed using Eq. [1].

## Data Availability

The statistical analyses were performed using R^35^. K-means clustering was conducted with the ‘h2o’ package^36^. For fitting the van Genuchten model, managing soil data, computing accuracy metrics, and visualizing the analysis results, we used the following R packages: DEoptim^37,38^, doParallel^39^, raster^40^, terra^41^, sf^42^, snowfall^43^, psych^44^, dplyr^45^, and ggplot2^46^. The codes used to develop the national soil hydrologic groups map are specific for the datasets and maps available in Hungary. However, they can be adapted for use with other datasets. The R scripts for the k-means analysis and the definition of expert-based rules are available on the Zenodo online repository of the maps^30^ (10.5281/zenodo.15223611).
